# Structural validity of the Pittsburgh Sleep Quality Index among medical students in Iran

**DOI:** 10.1038/s41598-024-51379-y

**Published:** 2024-01-11

**Authors:** Mohammad Reza Shadzi, Mahdi Rahmanian, Aigin Heydari, Alireza Salehi

**Affiliations:** 1 Department of MPH, School of Medicine, Shiraz University of Medical Sciences, Shiraz, Iran; 2 Cardiovascular Research Center, Shiraz University of Medical Sciences, Shiraz, Iran

**Keywords:** Circadian rhythms and sleep, Psychiatric disorders, Epidemiology

## Abstract

The Pittsburgh Sleep Quality Index (PSQI) is a self-reported questionnaire evaluating sleep quality based on seven domains of sleep disorders. However, the factor structure of PSQI needs to be well-established among medical students. This study was conducted to establish the factor structure of PSQI among medical students. Four-hundred and four medical students completed the PSQI. Considering each PSQI component as an "observed variable," exploratory factor analysis was conducted. Six models explaining the factor structure of PSQI were compared by confirmatory factor analysis to achieve the best model. All PSQI components were loaded on three factors. The first factor included sleep duration and habitual sleep efficiency, the second was subjective sleep quality and sleep latency, and the third was sleep disturbances, sleeping medications, and daytime dysfunction (root mean square error of approximation =  < 0.01, goodness of fit index = 0.99). The differences in Bayesian information criteria and root mean square error of approximation between our best-fit model and each of the other resulted in rejection of all other Models. Besides confirming the structural validity among medical students, our findings indicated the adequacy of the 3-factor structure of PSQI, including sleep efficiency, perceived sleep quality, and daily disturbances in this population.

## Introduction

The Pittsburgh Sleep Quality Index (PSQI) is a self-reported questionnaire, used to evaluate the sleep quality based on seven different domains of sleep disorders^1^. Since its invention, its different psychometric aspects have been assessed^2,3^. Nevertheless, there is no consensus about the scoring validity of the PSQI. It is necessary to assess the scoring validity, as a structural aspect of construct validity^4^. Scoring validity of the PSQI depends on the sleep pattern and characteristics of the targeted population^5^. A recent systematic review revealed that the factor structure of PSQI varied in different populations and called for more researches to be conducted for scoring validity of PSQI^6^.

Poor sleep quality is prevalent among medical students^7^. In southern Asia, poor sleep quality was also common among medical students. In Saudi Arabia, more than 50% of the medical students have experienced poor sleep quality^8^. The prevalence of poor sleep quality was 39% among medical students of Pakistan^9^. A considerable prevalence of sleep disorders such as using hypnotics, insomnia and undesirable sleep satisfaction has been reported among Iranian medical students^10^. Another study on medical students in Iran demonstrated that the prevalence of poor sleep quality was higher than normal population which might be attributed to higher rate of mental health problems in medical students as well as multiple and long hospital shifts as a standby doctor^11^.

Previous researches from medical students from Middle East region revealed substantial similarities in sleep prevalence, sleep patterns, and demographic factors (such as marital status)^9,11^. Besides, studies suggest that sleep habits of medical students from Middle East region were unsatisfactory compared to medical undergraduates from other parts of the world. As a simple example, a study on Pakistanian medical students showed that more than 70% of participants went to bed after midnight which was remarkably higher than studies on worldwide medical students^9^. On the other hand, Most of the studies evaluating the factor structure of PSQI among Asians are from south-east Asia^12,13^. Although a recent study in Saudi Arabia assessed the structural validity of PSQI in physicians, there cannot be attributed to medical students, as physicians’ life styles may differs vastly from medical students due to factors like lesser need to study and allocating more times to work^14^.

In total, there are few reports from Middle East region to assess the factor structure of PSQI and none of them has evaluated that among medical students. In addition, given the probable different patterns of sleep disorders among Asian medical students, factor structure of PSQI needs to be assessed among them. Hence, we aimed to conduct a study to establish the factor structure of PSQI among Iranian medical students.

## Methods

### Participants

The data presented in this article was provided by a cross-sectional study assessing the relationships between problematic internet use, mental health problems, and sleep quality among medical students of Shiraz University of Medical Sciences^11^. In that study, participants completed the Persian version of PSQI^15^ as a sub-questionnaire of a main questionnaire, from May to August 2018. Minimum sample size was calculated based on two distinct methodologies and statistical analysis which aimed to be conducted in that study. Firstly, for applicability of Structural Equation Modeling (SEM) analysis we needed at least 200 students to be participated in that study. Furthermore, to get more accurate results regarding the epidemiologic aspects of problematic internet use, mental health problems, and sleep quality, the following formula was used to calculate the minimum sample size, with the prevalence (p) of 0.42–0.5 for each main variables based on our pilot study on 36 participants, Z = 1.96 and Error Level (d) = 0.05:

$$n=\frac{{Z}^{2}p(1-p)}{{d}^{2}}$$

Therefore, the minimum sample size was calculated to be 384. At the time of the study, 1568 students were studying general medicine at Shiraz University of Medical Sciences. Predicting the missing data, better describing the population and to achieve the adequate sample size in that study, about one-third of the whole medical students were selected to participate in the study. The sampling population was stratified into three educational levels of basic sciences, physiopathology, and clinical stage. Then, sampling was done using a simple random sampling method based on the student identification number, in proportion to the size of each educational level.

The inclusion criterion was being a first- to seventh-year medical student. Accordingly, 487 medical students were asked to complete the questionnaire. Sixty-seven students refused to participate in the study, resulting in an 86.2% response rate. Sixteen students were excluded from the study, leaving the final sample of 404 students. Exclusion criteria were defined as follows.

A. Not answering one of the first four questions of PSQI (14 students), or
B. Not answering more than two questions of PSQI (2 students).

Because each of the first to four questions of the PSQI has a basic role in creating the domains of the global sleep quality, criterion A was defined to prevent data misanalysis.

This study was approved by the Ethics Committee of the Shiraz University of Medical Sciences (IR.SUMS.MED.REC.1397.102).

### Measure

PSQI includes 19 items summarized into seven components of "subjective sleep quality," "sleep latency," "sleep duration," "habitual sleep efficiency," "sleep disturbances," "sleeping medications," and "daytime dysfunction" after simple mathematical calculations^1^. The score of each component ranges from 0 to 3. The total PSQI score is calculated by summation of the scores of the seven components (ranging from 0 to 21). A score of more than six was considered to identify the students with poor sleep quality^15^.

### Statistical analysis

The sample characteristics were described using mean ± standard deviation (SD) or frequency based on the type of variables. Correlations between PSQI components were calculated by Spearman's correlation.

Descriptive statistics were followed by factor analysis. To obtain the optimal factor model, we randomly divided our study population into two non-equal samples. Firstly, exploratory factor analysis (EFA) was conducted on one-third of our total participants (144 students). Then, confirmatory factor analysis (CFA) was conducted on the remaining participants (260 students). This a statistically evident approach of doing factor analysis in a unique data set which allocates more data for CFA and also provided this study with the minimum sample size of 200 participants for CFA and adequate sample size for EFA which is assessed by Kaiser–Meyer–Olkin (KMO) index^16^.

KMO index and Bartlett's Test (BT) of sphericity were used to assess the applicability of EFA. The KMO acceptance level is for values higher than 0.5^17^. Then, EFA was conducted based on the eigenvalues of greater than 1, using the principal components analysis (PCA) method. Factor loadings were adjusted at greater than 0.5 to achieve accurate results. Lagrange modification indices were used to test whether any unmodeled paths significantly improved the model fit^18^.

CFA was conducted to achieve the best model. Based on the literature^1,5,12,19,20^ and the results of this study, six models explaining the factor structure of PSQI were compared in this study (Table 1). Each PSQI component was considered an "observed variable."

**Table 1:** Factor structure of six models assessed by Confirmatory Factor Analysis (CFA).

Component	Model A	Model B	Model C	Model D	Model E	Model F
1. Subjective sleep quality	1	1	1	1	1	1
2. Sleep latency	1	1	1	1	2	2
3. Sleep duration	1	2	2	2	3	3
4. Habitual sleep efficiency	1	2	2	2	3	3
5. Sleep disturbances	1	1	3	3	2	1
6. Use of sleep medication	1	1	1	3	2	2
7. Daytime dysfunction	1	1	3	3	1	1

Components with the same number are loaded into one factor.

Fit indices of root mean square error of approximation (RMSEA) < 0.05, minimum discrepancy divided by its degrees of freedom (CMIN/DF) < 2, chi-square test (χ^2^) p-value > 0.05, the goodness of fit index (GFI) > 0.95, adjusted goodness of fit index (AGFI) > 0.95, comparative fit index (CFI) > 0.95, normed fit index (NFI) > 0.95 and non-normed fit index (NNFI) > 0.95 were considered for acceptance of the model fit^21^. Since GFI, AGFI, CFI, NFI, and NNFI indices estimate the relative position of a model between the worst to the best model, higher amounts of them are preferred.

Three indices of χ^2^ statistics, consistent Akaike information criteria (CAIC) and Bayesian information criteria (BIC), were calculated to compare different models. When comparing the models, the preference is for lower amounts of χ^2^, BIC, and CAIC^22,23^. Also, if the difference between the BICs of two models is ten or more, the one with a higher BIC is rejected^23^. All the analysis was performed using SPSS Statistics and SPSS Amos, version 24.

Missing data presented in demographic variables (age, gender, marital status) were list-wised excluded. The highest amount belonged to age variable which had 3.7% missing data. The same strategy was used for dealing with the PSQI items. Missing data for each item were found to be less than 1% and item number seven had the maximum missing data (3 out of 404).

### Ethics approval and consent to participate

The privacy of the patients was protected. This study was approved by the Ethics Committee of the Shiraz University of Medical Sciences (IR.SUMS.MED.REC.1397.102), and written informed consent was obtained from the subjects. All methods were carried out in accordance with relevant guidelines and regulations.

## Results

### Descriptive information

The characteristics of the sample are reported in Table 2. The mean of PSQI scores was 6.18 (SD = 3.42, Cronbach's alpha = 0.70). The descriptive statistics and correlation matrix of the seven PSQI components are shown in Supplementary Table 1 online.

**Table 2:** Sample characteristics.

	Frequency (%)
Poor sleep quality	164 (40.6)
Sleep medications use	73 (18.1)
More than one hour sleep latency	68 (16.8)
Age in years, median (range)	22 (19–32)
Gender	
Male	198 (49.7)
Female	200 (50.3)
Marital status	
Single	372 (93.2)
Married	27 (6.8)

### Exploratory factor analysis

KMO index and BT showed an acceptable level of conducting EFA (KMO = 0.71, BT p-value < 0.001). The eigenvalue-based analysis suggested that all the observed variables can be loaded on two factors (see Supplementary Fig. 1 online): one factor represented sleep duration and habitual sleep efficiency, and another represented the other five variables (Model B). Factor loadings are shown in Supplementary Table 2 online. Lagrange modifications indices suggested that three variables of sleep disturbances, sleeping medication, and daytime dysfunction can be loaded on one independent factor. When we applied the command, the final model was configured as a 3-factor model (Model D).

### Confirmatory factor analysis

**Figure 1:**
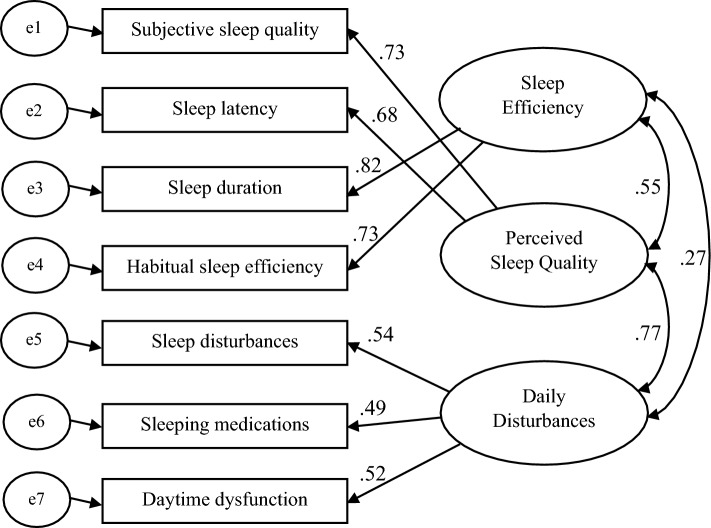
Model D: The best fitted model describing the factor structure of Pittsburgh Sleep Quality Index (PSQI); standardized path coefficients are presented.

Results of CFA revealed that Model D produced the best fit. As shown in Table 3, all fit indices of Model D were at the significant level of acceptance. The lowest amounts of the indices of χ^2^, CAIC, and BIC were produced by Model D (Table 3). Model A had the worst BIC, with a difference of more than 60 compared to the other models. The differences in BIC between Model D and each models of A, E, and F were more than 10 and only in Model D, the fit index of RMSEA was at the significant level of acceptance (< 0.01), leading to the rejection of all other models. Standardized path coefficients of Model D are shown in Fig. 1.

**Table 3:** Results of Confirmatory Factor Analysis (CFA): fit statistics of six models.

Index	Model A	Model B	Model C	Model D	Model E	Model F
RMSEA	0.16	0.06	0.05	< 0.01	0.06	0.07
CMIN/DF	7.19	1.88	1.48	1.02	2.05	2.18
GFI	0.90	0.97	0.98	0.99	0.98	0.97
AGFI	0.80	0.94	0.96	0.97	0.94	0.93
CFI	0.74	0.97	0.98	0.99	0.97	0.96
NFI	0.72	0.93	0.95	0.97	0.94	0.93
NNFI	0.61	0.94	0.97	0.99	0.94	0.93
χ^2^ (p-value)	100.73 (< 0.001)	24.48 (0.027)	16.23 (0.133)	11.17 (0.429)	22.58 (0.020)	23.72 (0.014)
CAIC	192.58	122.89	127.76	122.70	134.11	135.25
BIC	178.58	107.89	110.76	105.70	117.11	118.25

*RMSEA* root mean square error of approximation, *CMIN/DF* minimum discrepancy divided by its degrees of freedom, *GFI* goodness of fit index, *AGFI* adjusted goodness of fit index, *CFI* comparative fit index, *NFI* normed fit index, *NNFI* non-normed fit index, *χ*^*2*^ chi-square test, *CAIC* consistent Akaike information criteria, *BIC* Bayesian information criteria.

## Discussion

This study revealed the preference for the 3-factor model of PSQI among Iranian medical students. Our results revealed that two components of subjective sleep quality and sleep latency are loaded to perceived sleep quality. In comparison, three components of sleep disturbances, sleeping medications, and daytime dysfunction are loaded to the factor of daily disturbances. These results imply that sleep disturbances may trigger daytime dysfunction as well as using sleeping medications. However, none of these three components is separately related to subjective sleep quality.

Previous studies on sleep quality among medical students showed that there is a direct correlation between insomnia and subjective sleep quality. In contrast, parasomnia and subjective sleep quality are not associated^10^. Sleep latency- which reflects insomnia- plays the most crucial role in self-percept sleep dissatisfaction. This explains how two components of subjective sleep quality and sleep latency play an independent factor (perceived sleep quality) in the development of poor sleep quality.

There is consensus on this finding that sleep duration and habitual sleep efficiency are loaded into a single factor^5,12,19,20^. This structure is always seen even in a small population, no matter if PSQI produces a 2-factor or 3-factor structure. For example, the same pattern was yielded by Nicassio et al., based on 107 rheumatoid arthritis patients^24^. It may be due to an excellent convergent validity between sleep duration and habitual sleep efficiency, besides a good discriminative validity between their loading factor (sleep efficiency) and the other factors.

The factor structure of the preserved model is similar to the one yielded by Koh et al. based on a multi-ethnic Asian population study^12^. This model differs from the American 3-factor model, in which sleep medication is loaded on perceived sleep quality instead of daily disturbances^19^. Also, our model differed from African and South American college students' models^5^. It seems that the factor structure of PSQI produces a unique pattern among different Asian populations. However, some data show that the theory is not expressed as a definite conclusion^5,20^.

Our results differed from those of the Koh's et al. study^12^. First, Cronbach's alpha was higher in our study. The corresponding standardized path coefficients were also higher in our study. Besides, the Singapore Health study accepted the Cole et al. model^12,19^. In fact, this 3-factor structure of PSQI better fits the data obtained from medical students. This is probably due to the different sleep patterns of medical students and reminds the severity of sleep disorders among them^7^. In support of this point, a significantly higher prevalence of using sleep medications was also documented in this study.

In addition to determining the factor structure of PSQI, our results emphasizes on the severity of poor sleep quality in medical students. For example, in our study both sleep duration and using sleep medications variables predicted about 67% and 24% (squared multiple correlations) of changes of their loading factors, respectively. These amounts were considerably higher in comparison to other researches on various target populations^12,19^. Therefore, it is logical to devote more efforts and finances to preventing the predisposing factors of these domains to improve sleep quality in medical students. There are many factors such as drug abuse, alcoholism, depression, anxiety, problematic internet use, hospital shifts, and educational stress that might play more significant roles in medical students in suffering poor sleep quality; though, the differences in epidemiologic and pathophysiologic aspects of these factors in medical students in comparison to normal population is less documented in the literature. Hence, more researches should be conducted to clarify the role of these probable factors in predisposing medical students to poor sleep quality.

Altogether, studies showed a unique sleep pattern besides the similarity of other population characteristics among southern Asian medical students. An example of such a similarity can be seen in Surani's et al. study, where gender ratio, mean age, marital status, and prevalence of sleep quality (39.5%) were similar to our study^9^. Another study among Pakistanian medical students showed that the prevalence of more than one hour of sleep latency (18.9%) and using sleep medications (25.6%) was similar to this study^25^. Concluding, we recommend our 3-factor model of PSQI to be used for sleep quality assessment of southern Asian medical students.

### Limitations

This is the first study examining the factor structure of PSQI among Middle East region medical students. Due to a lack of facilities, we only assessed the factor structure of PSQI among medical students. Hence, the study's results might not be applied to other students.

## Conclusions

In conclusion, besides confirming the structural validity among medical students, our findings indicated the adequacy of the 3-factor structure of PSQI, including sleep efficiency, perceived sleep quality, and daily disturbances in this population. Further studies are recommended to clarify the factor structure of PSQI in different populations.

### Supplementary Information

Supplementary Information.

## Data Availability

The datasets used and/or analysed during the current study are available from the corresponding author on reasonable request.
