# Supplementary material for: Structural validity of the Pittsburgh Sleep Quality Index among medical students in Iran
Source: Sci Rep. 2024 Jan 11;14:1538. doi: 10.1038/s41598-024-51379-y (PMC10794382; doi:10.1038/s41598-024-51379-y)
Supplement: Supplementary file 1 — Supplementary Information. [file 41598_2024_51379_MOESM1_ESM.docx]

| Supplementary Table 1. Descriptive statistics and correlation matrix of Pittsburgh Sleep Quality Index (PSQI) components | | | | | | | | | |  |
| --- | --- | --- | --- | --- | --- | --- | --- | --- | --- | --- |
| Component | *M* | *SD* | 1. | 2. | 3. | 4. | 5. | 6. |  |  |
| 1. Subjective sleep quality | 1.17 | .76 |  |  |  |  |  |  |  |  |
| 2. Sleep latency | 1.33 | 1.01 | .47^**^ |  |  |  |  |  |  |  |
| 3. Sleep duration | .95 | 1.01 | .26^**^ | .25^**^ |  |  |  |  |  |  |
| 4. Habitual sleep efficiency | .43 | .79 | .23^**^ | .28^**^ | .51^**^ |  |  |  |  |  |
| 5. Sleep disturbances | 1.00 | .53 | .31^**^ | .28^**^ | .03 | .07 |  |  |  |  |
| 6. Use of sleep medication | .26 | .61 | .27^**^ | .27^**^ | .07 | .12^*^ | .29^**^ |  |  |  |
| 7. Daytime dysfunction | 1.05 | .91 | .34^**^ | .25^**^ | .09 | .20^**^ | .31^**^ | .27^**^ |  |  |
| *M*, mean; *SD*, standard deviation; * p< 0.05; ** p< 0.01 | | | | | | | | | | |


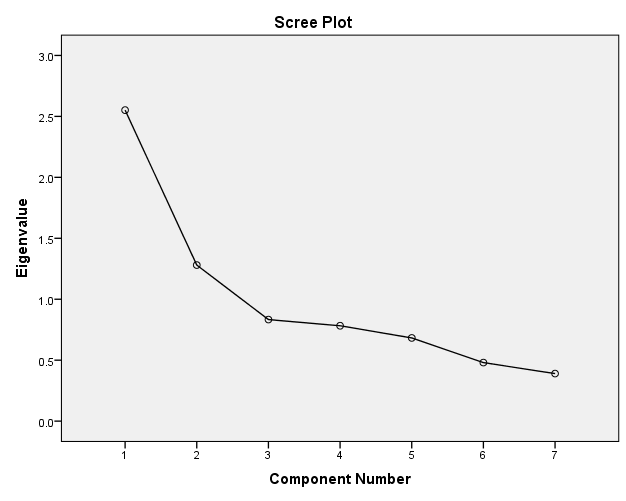


Supplementary Figure 1. The Scree Plot of Pittsburgh Sleep Quality Index (PSQI) by Exploratory Factor Analysis (EFA)

| Supplementary Table 2. Structure matrix of Pittsburgh Sleep Quality Index (PSQI) obtained from oblimin rotation | | |
| --- | --- | --- |
| Component | Factor 1 | Factor 2 |
| 1. Subjective sleep quality | 0.57 |  |
| 2. Sleep latency | 0.65 |  |
| 3. Sleep duration |  | - 0.85 |
| 4. Habitual sleep efficiency |  | - 0.83 |
| 5. Sleep disturbances | 0.70 |  |
| 6. Use of sleep medication | 0.65 |  |
| 7. Daytime dysfunction | 0.63 |  |
| Factor loadings between – 0.5 and 0.5 are not shown. | | |
